# Th17 cell-mediated immune response in a subpopulation of dogs with idiopathic epilepsy

**DOI:** 10.1371/journal.pone.0262285

**Published:** 2022-01-13

**Authors:** Anna Knebel, Annika Kämpe, Regina Carlson, Karl Rohn, Andrea Tipold

**Affiliations:** 1 Department of Small Animal Medicine and Surgery, University of Veterinary Medicine, Hannover, Germany; 2 Department of Biometry, Epidemiology and Information Processing, University of Veterinary Medicine Hannover, Hannover, Germany; Szegedi Tudomanyegyetem, HUNGARY

## Abstract

**Background:**

Canine idiopathic epilepsy (IE) is a common neurological disease with severe impact on the owner´s and the dog’s quality of life. A subpopulation of dogs with IE does not respond to antiseizure drugs (non-responder). Th17 cells (T helper cells) and their proinflammatory Interleukin-17 (IL-17) are part of the immune system and previous studies showed their involvement in the pathogenesis of several autoimmune diseases. Non-responder might have an abnormal immune response against structures of the central nervous system. To discover a new aetiology of canine IE and thereby optimising the therapy of intractable IE, this prospective study aimed to investigate Th17 cells and IL-17 in dogs with IE. The underlying hypothesis was that in some dogs with IE a Th17 cell-mediated immune response could be detectable.

**Methods:**

57 dogs with IE and 10 healthy dogs (control group, C) were enrolled in the study. EDTA blood was taken to measure Th17 cells by flow cytometry. IL-17 was measured in 35 cerebrospinal fluid (CSF) and 33 serum samples using an enzyme-linked immunosorbent assay (ELISA). It was investigated whether there was a significant increase of stimulated Th17 cells in blood samples or of IL-17 in serum and CSF samples of dogs with IE in comparison to C. Correlations between the amount of Th17 cells/μL or IL-17 and different clinical parameters e.g. seizure frequency, seizure type, seizure severity or treatment response were evaluated. Additionally, Th17 cells/μL were randomly controlled of 17 dogs with IE and were examined for changes over time and in relation to treatment response.

**Results:**

Ten dogs with IE had strongly elevated stimulated Th17 cells/μL within the blood (>100 Th17 cells/μL). A slight positive correlation between stimulated Th17 cells/μL and seizure severity (p = 0.046; rSpear = 0.27) was proven in these dogs. In addition, 4/10 dogs with elevated Th17 levels experienced cluster seizures and status epilepticus in comparison to 9% of the dogs with non-elevated Th17 levels (<100 Th17 cells/μL). Dogs with IE had significantly higher IL-17 values in CSF and serum samples compared to C (p<0.001; p<0.002; respectively).

**Conclusion:**

In single dogs with IE, strongly increased amounts of Th17 cells were detectable and dogs with elevated Th17 cells seemed to have a greater risk for experiencing a combination of cluster seizures and status epilepticus. Therefore, an underlying Th17-cell mediated immune response was suspected and hence anti-inflammatory drugs could be indicated in these single cases with intractable epilepsy.

## Background

Epilepsy, the most common chronic brain disease in dogs, is defined as the enduring predisposition of generating two or more unprovoked seizures at least 24 h apart [1–4]. The disease is affecting quality of life, not only for the dog but also for the owner [5]. Idiopathic epilepsy (IE) is divided by the „International Veterinary Epilepsy Task Force” (IVETF) into three groups: genetic epilepsy (a causative gene has been identified), suspected genetic epilepsy (a causative gene has not been identified, but there is a high prevalence in the breed (>2%)) and epilepsy of unknown cause [1]. To date, IE is still a diagnosis of exclusion [6, 7] and reactive seizures (RS) [1, 8] as well as structural changes of the brain (structural epilepsy; SE) have to be ruled out as a cause for seizures [1, 6]. A part of dogs with IE do not respond to treatment with antiseizure drugs (ASD) [9, 10] or have a severe phenotyp such as cluster seizures (CS) and status epilepticus (StEp) [1, 11, 12]. Th17 cells (CD3+CD4+IL-17+) which are part of the immune system and emerge next to Th1 (CD3+CD4+INF-y+) and Th2 cells (CD3+CD4+IL-4+) from naive T helper cells [13–16], play an important role in protection against pathogens and are importantly implicated in the pathogenesis of autoimmune disorders [17–21]. They are defined by their capacity to secrete the proinflammatory Interleukin-17 (IL-17) and assume a vital role in activating and modulating immunological or non-immunological cells [14, 19, 21–27]. In humans, Th17 cells and IL-17 were shown to promote autoimmune diseases, such as rheumatoid arthritis, ankylosing spondylitis, multiple sclerosis, amyotrophic lateral sclerosis, systemic lupus erythematosus and inflammatory bowel disease [28–36]. Vezzani et al. discussed those inflammatory processes within the central nervous system (CNS) and their role in the pathophysiology of seizures [37, 38]. Furthermore, autoimmune-mediated forms of epilepsy were firstly described as idiopathic [39–43]. Supporting the theory about an inflammatory involvement in a subpopulation of patients, who do not respond to ASD, but clinically improved after application of immune-modulatory drugs, steroids or NSAIDs (non-steroidal anti-inflammatory drugs) [43–50]. In veterinary medicine, Freund-Revilla et al. investigated a Th17-skewed immune response in canine steroid-responsive meningitis-arteritis (SRMA). In a control group of dogs with IE, IL-17 spot-forming cells were detected [51]. Additionally, it was demonstrated that Th17 cells are present and measurable by flow cytometry in the blood of healthy dogs and tissues of dogs with chronic idiopathic diseases [52, 53]. Therefore, in the current study the occurrence of Th17 cells were investigated thoroughly in dogs with IE. This prospective study hypothesised that an underlying Th17 cell-mediated response in a subpopulation of dogs with IE is detectable and can be correlated to clinical findings such as seizure severity and response to treatment.

## Materials and methods

### Study population

This prospective study was approved by the Animal Care Committee of the Government of Lower Saxony and national regulations for animal welfare (experiment number 33.8-42502-05-18A290) and was performed with the owner’s informed written consent. From 1 December 2017 to 30 August 2018 57 patients were enrolled at the Department of Small Animal Medicine and Surgery, University of Veterinary Medicine Hannover, Germany and included in the current study if they matched at least the IVETF TIER I and TIER II confidence level for the diagnosis of IE [6]. Dogs, which met the IVETF TIER II confidence level, were preferred. If dogs were at the time point of seizure onset younger than 6 months or older than 6 years of age and IE was suspected, MRI (Magnetic resonance imaging) and CSF (cerebrospinal fluid) analysis had to be performed to rule out SE. Evaluation of dogs was performed by a Resident or a Diplomate of the European College of Veterinary Neurology (ECVN). Information on the dog´s signalement, seizure type, seizure severity, information about ASD, date of the last seizure day before blood taking/CSF tap, the number of seizure days the month before blood taking/CSF tap and the number of seizure days six months before blood taking/CSF tap was collected. Ten healthy clinic-owned dogs (beagles) served as controls (C).

### Blood and cerebrospinal fluid samples

For the measurement of Th17 cells by flow cytometry, EDTA (ethylenediaminetetraacetic acid) blood was taken from 57 dogs with naturally occuring IE and 10 healthy beagles via puncture of the cephalic or saphenous vein and stored overnight at room temperature in the dark [53]. The amount of blood varied from 1–5 mL depending on the size and clinical status of the dog and was documented for later calculation of the absolute number of Th17 cells. Numbers of lymphocytes (x10^3^/μL) in the blood were analysed using the Advia2120i Hematology System (Advia2120i Hematology System Siemens Healthcare GmbH, Eschborn, Germany). To measure IL-17, CSF samples were collected by suboccipital puncture when dogs were under general anesthesia. In dogs with IE, the CSF puncture was done after performing the MRI examination (Phillips Achieva Medical Systems, Eindhoven, The Netherlands) of the brain for routinely exclusion of structural changes of the brain as the cause for seizures. General anesthesia was induced with intravenous levomethadone (0.2 mg/kg), diazepam (0.43 mg/kg) and propofol (6.5 mg/kg). After intubation, anesthesia was maintained with isoflurane in air and oxygen. Serum samples were taken at the same time and both samples were stored at -20°C. All dogs recovered well after anesthesia and in dogs with IE, antiseizure treatment was started.

### Measurement of Th17 cells using flow cytometry

Based on the previously published modified protocol by Knebel et al. [53], peripheral blood mononuclear cells (PBMC) were isolated from blood samples, that had been stored overnight, using Lymph 24+ Spin Medium (pluriSelect Life Science UG & Co. KG, Leipzig, Germany). Briefly, isolated cells were stained with mouse anti-dog CD8 alpha (1:11; Bio-Rad Laboratories, Inc., Irvine, CA, USA), mouse anti-dog CD11b (1:11; Bio-Rad Laboratories, Inc.) and mouse anti-canine CD21 (1:11; Bio-Rad Laboratories, Inc.). Human TruStainFcX™ (1:20; BioLegend ®, San Diego, CA, USA) was used to block the Fc receptors on cells. By using goat anti-mouse IgG MicroBeads (1:5; Miltenyi Biotec GmbH, Bergisch Gladbach, Germany) and the autoMACS® Pro Separator (Miltenyi Biotec GmbH, Germany), an enrichment of CD3+CD4+ cells by depletion of CD8alpha+, CD11b+ and CD21+ cells was achieved. After manual cell counting, gained CD3+CD4+ cells were resuspended in 400 μL of a specific lymphocyte medium (RPMI Medium1640 (Gibco™ life technologies Ltd., UK) with 5% FBS (foetal bovine serum; CytoGen GmbH, Greven, Germany), 1% HEPES solution (1M; Sigma-Aldrich®, Sigma-Aldrich Chemie GmbH) and 1% PenStrep (100 U/L Penicilin-G and 100 μg/mL Streptomycin; Sigma-Aldrich®, Sigma-Aldrich Chemie GmbH), the cell suspension was divided into two equal parts (200 μL each) and stored overnight within a cell culture plate (96-hole microplate, cellGrade TM-BRANDPlates®, BRAND GmbH + Co. KG, Wertheim, Germany) at 37°C and 5% CO_2_ (incubator, Type INDUCELL 55, MMM Medcenter Einrichtungen GmbH, Planegg, Germany). The next day, one part of the cell population was stimulated by adding 200 μL of a stimulation medium containing PMA (Phorbol-12-myristat-13-acetat; 25 ng/mL; Antibodies-online, Aachen, Germany) and ionomycin (in vitro stimulation; Ionomycin calcium salt (500 ng/mL; Sigma-Aldrich®, Sigma-Aldrich Chemie GmbH) and incubated for 6 hours. The other cell suspension was used as a negative control. After three hours, Brefeldin A (1 μg/mL; Sigma-Aldrich®, Sigma-Aldrich Chemie GmbH, Germany) was added to both cell populations. Because Th17 cells are a rare cell population, stimulation was recommended for better interpretation of results [52, 53]. For exclusion of dead cells, staining with Viobility™ 405/520 Fixable Dye (1:100; Miltenyi Biotec GmbH, Bergisch Gladbach, Germany) was performed. Before further cell staining, Fc receptors were blocked with Human TruStainFcX™ (1:20; BioLegend®, San Diego, CA, USA). Then, the cell suspensions were divided into three equal parts (50 μL each) and cells of two cell suspensions were phenotyped using following antibodies: rat anti-canine CD4 PE-Cyanine7 (1:11; eBioscience, Inc., San Diego, CA, USA) and mouse anti-dog CD3 FITC (1:11; Bio-Rad Laboratories Inc., Irvine, CA, USA). Incubation with staining buffer alone served as native sample. For intracellular staining, cells were permeabilised with 0.03% saponin buffer and stained with biotinylated mouse anti-human IL-17A-Biotin antibody (50 μL; pre-dilution 1:100 in saponin buffer; Clone: 403D100.01/mAb5 Biotin conjugated; Dendritics SAS, Lyon, France). 50 μL of diluted Biotin mouse IgG1 kappa Isotype (pre-dilution 1:200 in saponin buffer; BioLegend ®, San Diego, CA, USA) was added to serve as a control (isotype control) and the same amount of saponin buffer was added to the native sample. APC streptavidin (pre-dilution 1:100 in saponin buffer; Allophycocyanin streptavidin; BioLegend®, San Diego, CA, USA) was added to the cell suspensions and served as a second step reagent. A multicolor flow cytometer (MACSQuant® Analyzer 10; Miltenyi Biotec GmbH) was used to identify and quantify the cells [53].

### Measurement of IL-17 using ELISA

IL-17 concentrations were determined in 35 CSF and 33 serum samples of dogs with IE and ten healthy dogs using a validated ELISA commercial kit for measuring IL-17 (SEA063CA-96, Cloud-Clone Corp., Houston, USA) following the manufacturer’s protocol [51, 54].

### Analysis methods

MACSQuantify™ software (Miltenyi Biotec GmbH, Bergisch Gladbach, Germany) was used to analyse the results of flow cytometry. Based on described gating strategies [53], dead cells and doublets were excluded and lymphocytes were gated by morphology. Within this cell population of lymphocytes, CD4+CD3+ positive cells were selected and gated. Within this population, Th17 cells were calculated in percent. The limit was set in the “isotype control” for approximately 2% positive cells (range: 1.9–2.1, [53]). Absolute values of Th17 cells were calculated in Th17 cells/μL. The values of Th17 cells/μL were then compared between the dogs with IE and C. On basis of the maximum measured level of Th17 cells in C, two groups of dogs with IE were created. The first group included dogs with IE and less than 100 Th17 cells/μL as detected in the control group and the second group of dogs with IE comprised dogs with higher amounts of Th17 cells (>100 Th17 cells/μL). Data were collected and processed using Microsoft® Excel 2016 (Microsoft Corporation, Redmond, WA, USA). Data were analysed for normal distribution using the Shapiro-Wilk-test. T-test was used for normal distributed data and the Wilcoxon Two-Sample Test for non-normal distributed data. It was investigated whether there were differences in the amount of Th17 cells and IL-17 in dogs with IE in comparison to dogs of the control group as well as whether there were correlations between Th17 cells and IL-17 and investigated clinical parameters (see Table 1). For the latter, Spearman´s correlation was used and the Fisher´s exact test was used to compare frequencies of qualitative single clinical parameters within different groups. SAS® Enterprise Guide 7.1 (SAS Institute, Cary, NC, USA) was used for statistical evaluation. p≤0.05 was considered statistically significant.

**Table 1:** Definition of investigated clinical parameters.

clinical parameter	definition	
**SF/M**: seizure days/month (seizure frequency)	number of counted seizure days one month before blood drawing/CSF tap	
**SF/6M**: mean seizure days/6months (seizure frequency)	mean of seizure days the last six months before blood drawing/CSF tap	
	**group**	**definition**
**ST:** seizure type	**1**	dog experienced generalized seizures
	**2**	dog experienced generalized and/or focal seizures
**SS:** seizure severity	**1**	dog experienced single seizure events
	**2**	dog experienced cluster seizures (CS)
	**3**	dog experienced status epilepticus (StEp)
	**4**	dog experienced CS and StEp
**TR:** treatment response	**1**	no ASD was administered so far
	**2**	good seizure control: seizure free one month before blood drawing/CSF tap and at least one ASD was administered
	**3**	insufficient seizure control: at least one seizure day one month before blood drawing/CSF tap and one ASD was administered
	**4**	poor seizure control: at least one seizure day within one month before blood drawing/CSF tap and at least two ASD were administered
**A-B:** period from last seizure day to blood drawing/CSF tap	**1**	The period lasted about: <24 hours
	**2**	The period lasted about: >24 hours-6 days
	**3**	The period lasted about: 7 days-3 weeks (21 days)
	**4**	The period lasted about: >3 weeks (>21 days)
**D:** duration of the disease (period from first seizure day to blood drawing/CSF tap)	**1**	The period lasted about: 1st day-6 months
	**2**	The period lasted about: 7 months-24 months
	**3**	The period lasted about: >25 months
**A:** age at seizure onset (in months)	**1**	6 months—36 months*
		*one dog was at seizure onset younger than 6 months, but IE was strongly suspected and MRI and CSF analysis were unremarkable
	**2**	37 months-72 months
	**3**	>72 months
**number of ASD**	number of counted ASD which had been administered to the patient at the time point of blood drawing/CSF tap (applicated ASD: phenobarbital, potassium bromide, imepitoin and levetiracetam as long-term therapy)	

### Follow-up measurement of Th17 cells

After three to seven months, Th17 cells were controlled randomly in 17 dogs with IE. Using seizure diaries, the average of seizure days of the last three months before the first blood drawing (date of measurement 1; **DM1**) and the follow-up examination/second blood drawing (date of measurement 2; **DM2**) was determined. Levels of Th17 cells changes over time and the association between the amount of Th17 cells with an increase or decrease of seizure days was calculated [7, 55]. Depending on the changes, the dogs were divided into two groups: **Group A** contained the dogs with a decreased mean of seizure days three months before the second blood drawing (DM2) or no seizure days in comparison to the mean of seizure days of DM1. **Group B** contained the dogs with an unchanged or increased mean of seizure days three months before the second blood drawing (DM2) in comparison to the mean of seizure days of DM1.

## Results

### Study population

In total, 57 client-owned dogs with IE fulfilled the criteria and were enrolled in the study and ten healthy university-owned beagles of the University of Veterinary Medicine Hannover, Germany, were used as a control group (C). The study population of dogs with IE consisted of 49 purebred dogs and eight crossbreed dogs (14%). The group of purebred dogs involved 14% Labrador Retriever (n = 8), 12% Australian Shepherd (n = 7) and two dogs (3%) each of the following breeds: Border Collie, German Shepherd, Rhodesian Ridgeback, Huskie, Bolonka Zwetna, Beagle and Maltese. With one dog per breed (2%) the following breeds were also represented: German Shorthair, Elo, Dogo Argentino, Belgian Shepherd Dog, Samoyed, Tibetan Terrier, Pug, Miniature Schnauzer, English Bulldog, Boston Terrier, Shiba Inu, Boxer, Landseer, Havanese, Tervueren, German Mittelspitz, Border Terrier, Pekingese, Golden Retriever, Wire-haired Dachshund, Dalmatian and English Springer Spaniel. The study population consisted of 12% females, 19% females neutered, 41% males and 28% were neutered males. In 82.5% of the dogs with IE, MRI and CSF analysis were evaluated. Within C, ten clinic-owned beagles (2–3 years of age) were included and consisted of eight male and two female dogs for Th17 cell measurement. For IL-17 measurement, male and female dogs were equally distributed.

### Results of the investigation of Th17 cells using flow cytometry in dogs with IE

**Fig 1:**
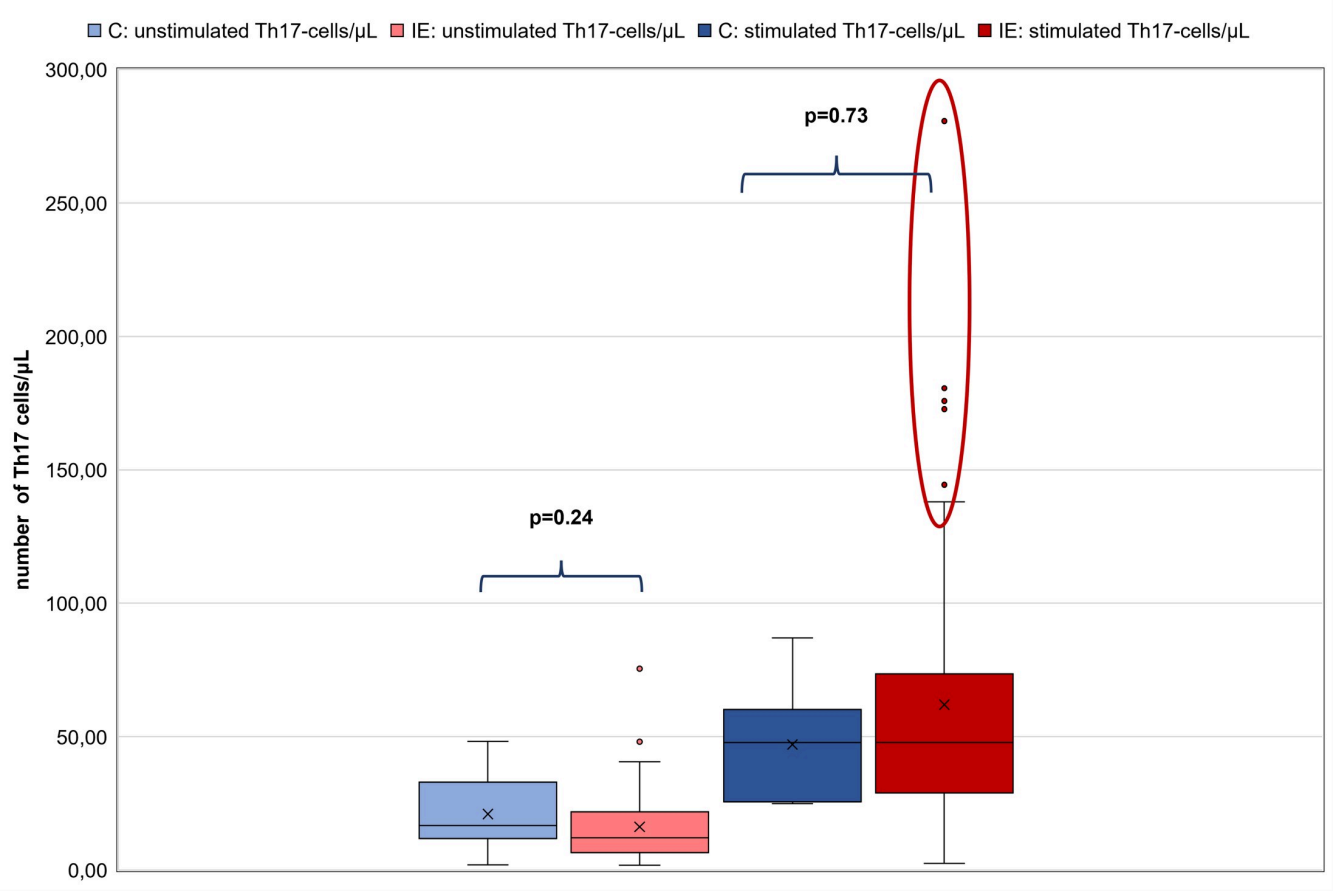
Comparison of the control group with dogs with IE regarding their amount of Th17 cells/μL. Left pale blue box plot showing results of unstimulated Th17 cells/μL of the control group (C) (n = 10) and the second pale red box plot showing results of unstimulated Th17 cells/μL of dogs with IE (n = 57). The third dark blue box plot showing results of stimulated Th17 cells/μL of C (n = 10) and the fourth dark red box plot showing results of stimulated Th17 cells/μL of dogs with IE (n = 57). All box plots represent the maximum, the third quartile, the mean, the median, the first quartile and minimum of the measured Th17 cells/μL. Dots represent outliers. There is no significant difference between the dogs of C in comparison to the dogs with IE regarding their amount of unstimulated as well as stimulated Th17 cells/μL (p = 0.24; p = 0.73, respectively). But analysis demonstrates that there are some outliers (demonstrated within the red circle) in the population of dogs with IE with strongly increased numbers of Th17 cells/μL. Abbreviations: C: control group; IE: idiopathic epilepsy.

**Fig 2:**
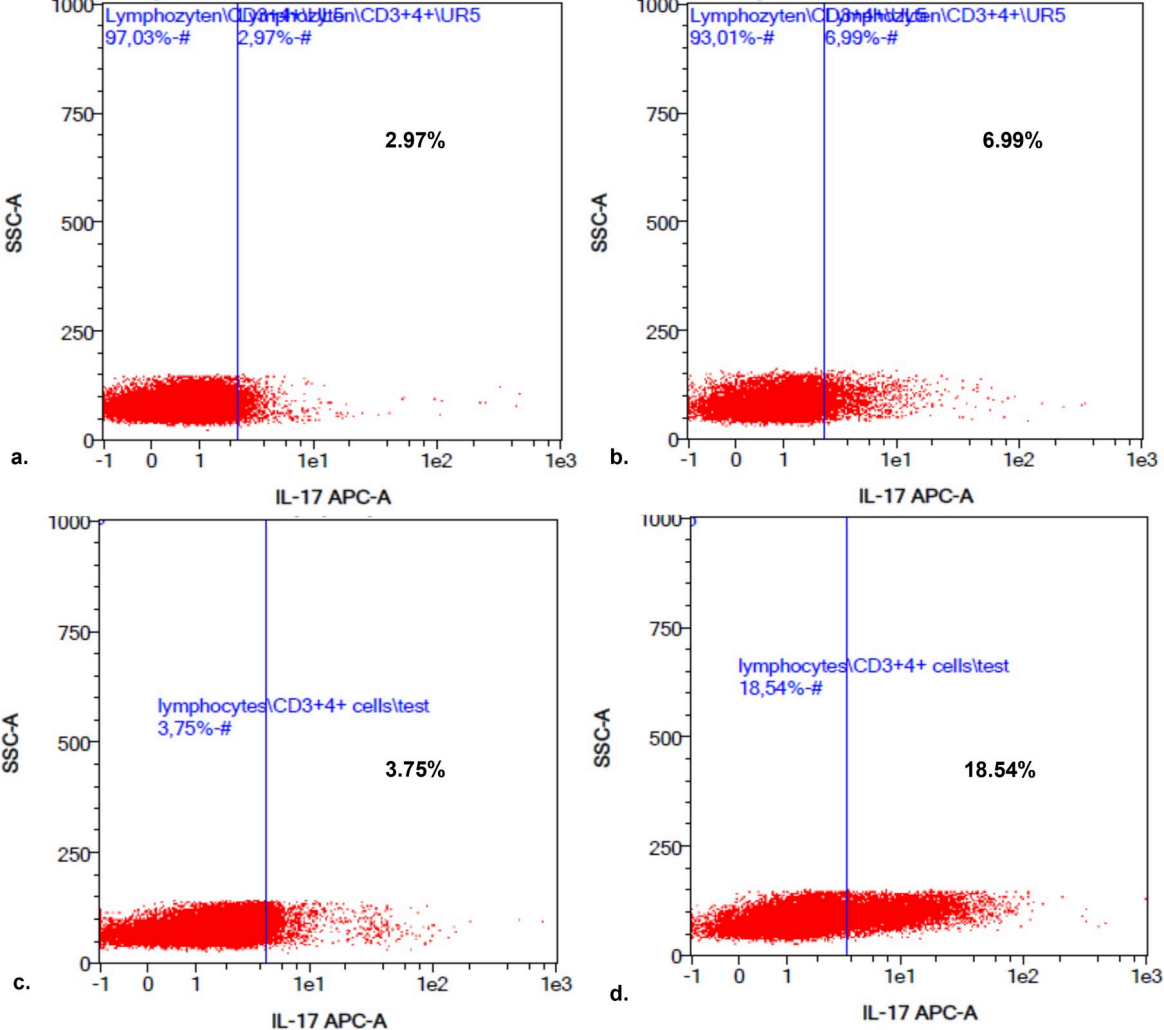
Presentation of a Th17 cell analysis after flow cytometry by MACSQuant Analysis Software of a healthy dog in comparison to a dog with IE. The pictures represent the results of Th17 cell analysis using MACSQuant analysis software (a.-d.). The left-sided upper box (a.) showing percentage of unstimulated Th17 cells in percentage of one dog of C, the left-sided lower box (c.) showing percentage of unstimulated Th17 cells in percentage of one dog with IE. The right-sided boxes (b. and d.) showing corresponding stimulated Th17 cells in percentage of the dogs of a. and c. Therefore, box b. showing the stimulated Th17 cells in percentage of the dog of C and d. of the dog with IE (c.). There is no big difference in the number of unstimulated Th17 cells (2.97% versus 3.75%) but in the amount of stimulated Th17 cells of dogs with IE in comparison to the C (6.99% versus 18.54%). Caution should be given that results of the MACSQuant analysis software were displayed in percentage. For comparative analyses absolute numbers of Th17 cells/μL were used. Abbreviations: C: control group; IE: idiopathic epilepsy.

No significant difference between unstimulated Th17 cells/μL of C (n = 10; 1.96–48.2 (mean: 21) Th17 cells/μL) and dogs with IE (n = 57; 1.84–75.41 (mean: 16.16) Th17 cells/μL; p = 0.24) and no significant difference between stimulated Th17 cells/μL of the C (24.9–87.03 (mean 47) Th17 cells/μL) and dogs with IE (2.54–280.74 (mean: 61.95) Th17 cells/μL; p = 0.73) were detected. However, it was strikingly noticeable that single individuals within the group of dogs with IE had higher numbers of stimulated Th17 cells/μL (outliers; Fig 1). Based on the maximum number of 87 stimulated Th17 cells/μL, that were found in C, ten dogs with IE had strongly increased numbers of stimulated Th17 cells/μL (>100 Th17 cells/μL). Therefore, the group of 57 dogs with IE was divided into two groups. The first group (**group 1; n = 47**) had 1.84 to 32.26 (mean 12.8) unstimulated Th17 cells/μL and 2.54 to 92.8 (mean 41.56) stimulated Th17 cells/μL and the second group (**group 2; n = 10**) included dogs, which had 2.35 to 75.41 (mean 31.81) unstimulated Th17 cells/μL and significantly increased numbers of stimulated Th17 cells/μL (mean 157.77; range 114.25–280.74), especially in comparison to the C (Fig 2).

Correlations between unstimulated and stimulated Th17 cells as well as the quotient of unstimulated and stimulated Th17 cells/μL of dogs with IE and the investigated clinical parameters (Table 1) were calculated. There was a slight positive correlation between stimulated Th17 cells/μL and seizure severity (p = 0.046; rSpear = 0.27), but no further correlations were detected. There were no statistically differences relative to the investigated parameters within group 1 and group 2. In comparison, dogs of group 1 had on average more seizure days one month as well as six months before blood drawing (SF/M and SF/6M; see Table 2). Generalized epileptic seizures were overrepresented in both groups (see Table 2) and regarding seizure severity approximately 74% of all dogs with IE had CS and about 18% had a StEp. 40% of dogs with strongly elevated Th17 cells (group 2) experienced CS and StEp in combination, in comparison to 9% of dogs within group 1 (see Table 2). There were no big differences in treatment response (TR), period from last seizure day to blood drawing (A-B) or duration of the IE (D) between both groups (see Table 2). About the half of the dogs of group 1 experienced the first seizure when they were 6–36 months old, in comparison to dogs of group 2, in which they were slightly older (37–72 months of age, see Table 2). Finally, in direct comparison, more dogs of group 2 needed more ASD, without reaching statistically significance (see Table 2).

**Table 2:** Comparison of group 1 (dogs with IE and <100 Th17 cells/μL; n = 47) and group 2 (dogs with IE and >100 Th17 cells/μL; n = 10) in relation to the investigated clinical parameters.

clinical paramater	subgroups	group 1 (dogs with IE and < 100 Th17 cells/μL; n = 47)		group 2 (dogs with IE and > 100 Th17 cells/μL; n = 10)	
**SF/M**		2.5 (mean); range: 0–29		1.5 (mean); range 0–4	
**SF/6M**		1.4 (mean); 0–13		0.78 (mean); range: 0–2.7	
		**n =**	**%**	**n =**	**%**
**ST**	**1**: generalized seizures	29	62	8	80
	**2**: experienced generalized and/or focal seizures	18	38	2	20
**SS**	**1**: single seizure events	12	25	1	10
	**2**: cluster seizures (CS)	29	62	5	50
	**3**: status epilepticus (StEp)	2	4	-	-
	**4**: CS and StEp	4	9	4	40
**TR**	**1**: no ASD was administered so far	8	17	2	20
	**2**: good seizure control	8	17	2	20
	**3**: insufficient seizure control	17	36	3	30
	**4**: poor seizure control	14	30	3	30
**A-B**	**1**: The period lasted about: <24 hours	12	26	3	30
	**2**: The period lasted about: >24 hours-6 days	10	21	2	20
	**3**: The period lasted about: 7 days-3 weeks (21 days)	10	21	2	20
	**4**: The period lasted about: >3 weeks (>21 days)	15	32	3	30
**D**	**1**: The period lasted about: 1st day-6 months	13	28	2	20
	**2**: The period lasted about: 7 months-24 months	18	38	3	30
	**3**: The period lasted about: >25 months	16	34	5	50
**A**	**1**: 6 months-36 months	25	53	3	30
	**2**: 37 months-72 months	16	34	5	50
	**3**: >72 months	6	13	2	20
**number of ASD**	no ASD	8	17	2	20
	1 ASD	23	49	3	30
	2 ASD	10	21	3	30
	3 ASD	6	13	2	20

### Follow-up measurement of Th17 cells

In nine dogs the seizure frequency improved (group A) and eight dogs suffered from deterioration of their seizure frequency (group B). There was no significant difference between the amount of Th17 cells of DM1 and DM2 (p = 0.42). Statistically, there were also no correlations between an increase or decrease of the Th17 cells and an increased or decreased seizure frequency (group A: p = 0.35; group B: p = 0.77). Nevertheless, a tendency of decrease of Th 17 cells with seizure improvement was seen because the amount of stimulated Th17 cells was lower in group A at DM2 (8.94–101.37 (mean: 45.47) Th17 cells/μL) in comparison to DM1 (14.4–135.79 (mean: 62.81) Th17 cells/μL) and higher in group B at DM2 (7.43–286.71 (mean: 81.80) Th17 cells/μL) in comparison to DM1 (20.95–180.57 (mean: 74.05) Th17 cells/μL).

### Results of the investigation of IL-17 values in pg/mL in CSF and serum samples using ELISA

**Fig 3:**
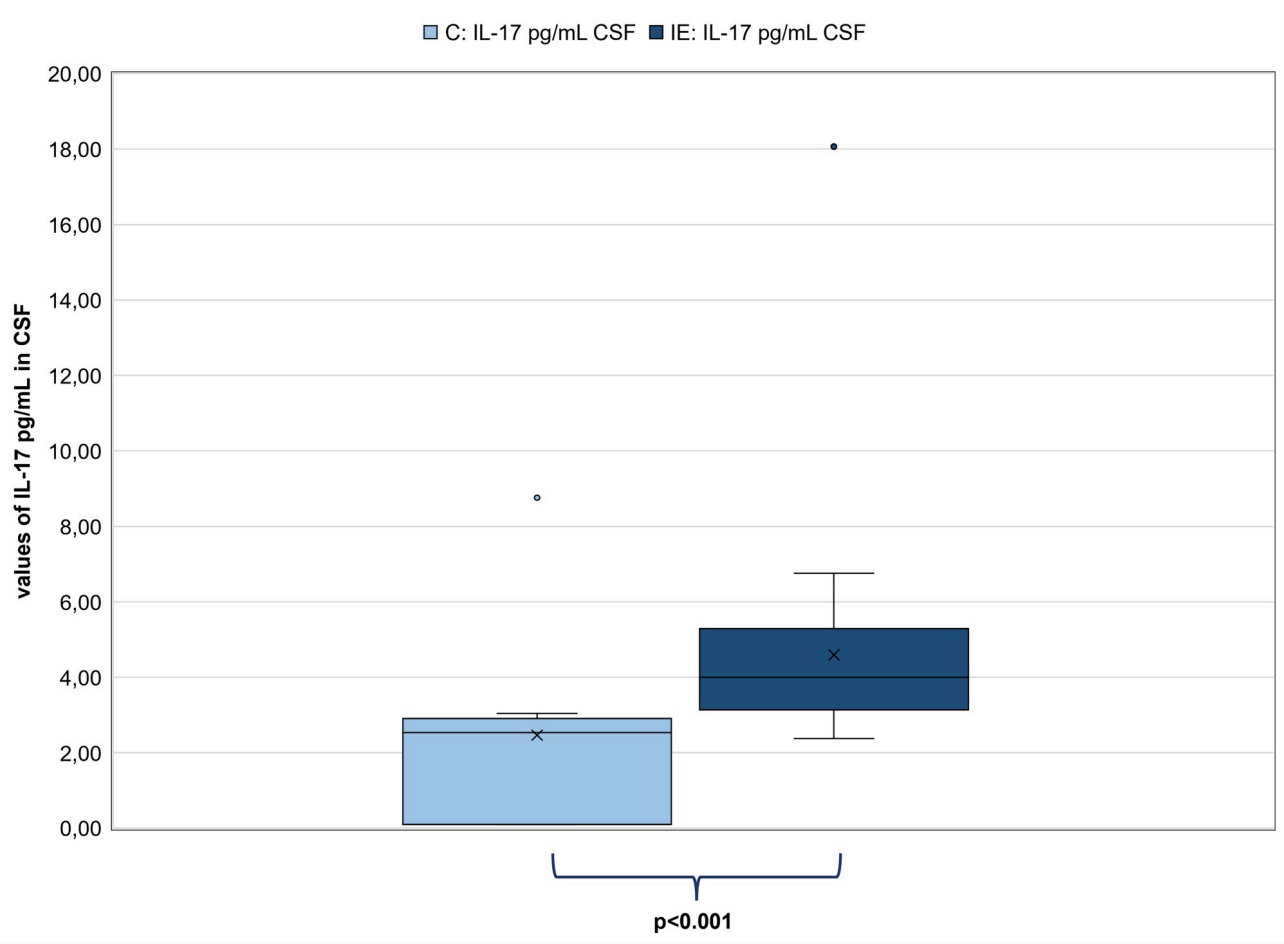
Comparison of the IL-17 values in pg/mL in CSF between dogs with IE and the control group (C). Left pale blue box plot showing results of IL-17 values of C (n = 10) and the right dark blue box plot showing results of IL-17 values of dogs with IE (n = 35). Both box plots represent the maximum, the third quartile, the mean, the median, the first quartile and minimum of measured IL-17 in CSF (in pg/mL). The IL-17 values of dogs with IE were significantly higher than those of C (p<0.001). Abbreviations: C: control group; CSF: Cerebrospinal fluid; IE: idiopathic epilepsy.

**Fig 4:**
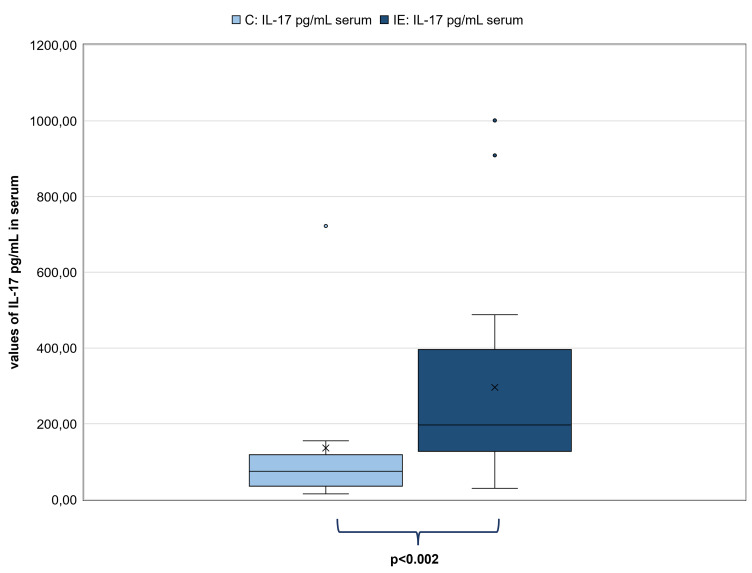
Comparison of the IL-17 values in pg/mL in serum samples between dogs with IE and the control group (C). Left pale blue box plot showing results of IL-17 values of C (n = 10) and the right dark blue box plot showing results of IL-17 values of dogs with IE (n = 33). Both box plots represent the maximum, the third quartile, the mean, the median the first quartile and minimum of measured IL-17 in serum samples (in pg/mL). The IL-17 values of dogs with IE were significantly higher than those of C (p<0.002). Abbreviations: C: control group; IE: idiopathic epilepsy.

There were neither correlations between IL-17 values of CSF samples and unstimulated and stimulated Th17 cells/μL (p = 0.19; p = 0.24 respectively) nor between IL-17 values of serum samples and unstimulated and stimulated Th17 cells/μL (p = 0.20; p = 0.98 respectively). There was also no correlation between IL-17 values of CSF samples and IL-17 values of serum samples of dogs with IE (n = 32; p = 0.67). Certainly, IL-17 values in CSF samples (n = 35) were significantly higher in dogs with IE (mean: 4.59 pg/mL; range: 2.37–18.06 pg/mL) in comparison to C (n = 10; mean: 2.47 pg/mL; range: 0.10–8.76 pg/mL; p<0.001; Fig 3). In addition, IL-17 values in serum samples were also significantly higher in dogs with IE (n = 33; mean: 296.14 pg/mL; range: 29.47–1001.00 pg/mL) in comparison to C (n = 10; mean: 135.86 pg/mL; range: 14.99–722.27 pg/mL; p<0.002; Fig 4).

Finally, there were no correlations between IL-17 values of serum samples and investigated clinical parameters but a slightly negative correlation between IL-17 values of CSF samples and the number of ASD (p = 0.04, rSpear = -0.34).

## Discussion

Inappropriate regulation of Th17 cells and IL-17 may lead to excessive proinflammatory cytokine release, which in turn can lead to chronic inflammatory and severe autoimmune diseases [17, 19, 21, 56]. In veterinary medicine, IL-17 is also becoming increasingly important in chronic idiopathic inflammatory processes [52] and autoimmune diseases [51]. To our knowledge, there is no study that measured Th17 cells in dogs with IE so far. In the current study, strikingly, ten dogs with IE and strongly elevated Th17 cells (17.54%) were detected, supporting the findings of Freund-Revilla et al. that single dogs with IE might have an autoimmune component [51]. Even though, the number of seizure days per se did not seem to be responsible for increased Th17 cells in the blood and vice versa, seizure severity slightly correlated with the amount of stimulated Th17 cells of dogs with IE because more dogs with strongly elevated Th17 cells experienced cluster seizures and status epilepticus, whereas cluster seizures per se did not lead to an upregulation of Th17 cells. In human medicine, autoimmune components in epilepsy are mostly accompanied with focal seizures and therapy-resistant epilepsies [41–43, 57–60]. In contrast to that, only 20% of dogs with IE and increased Th17 values (group 2) had generalized seizures and/or focal seizures in the current study. But similar to human medicine, unfortunately many dogs are still suffering from epileptic seizures, especially from cluster seizures and status epilepticus, despite antiepileptic treatment. Therapeutically, in humans, some patients with autoimmune forms of epilepsy responded better to immunotherapy or anti-inflammatory treatment options [43, 44, 46–50], NSAIDs [45] or treatment inhibiting the differentiation and amplification of Th17 cells or neutralizing IL-17 [61] than to ASD. Such treatment options could also be introduced in therapy regimens of dogs with elevated Th17 cells and intractable IE. Most dogs with strongly elevated Th17 values, experienced the first seizure at the age of 3–6 years. However, in human medicine, children and adolescents are described to be at higher risk for developing immune-mediated epilepsy [50, 59, 62–64]. In accordance with human studies, dogs with IE had significantly increased IL-17 values in CSF and serum samples [65] but in contrast, correlations between IL-17 values and seizure frequency or severity could not be detected in the current canine population [65–67]. In accordance with Mao et al. [65] no correlation between IL-17 and the duration of the disease was visible. In addition, no change in Th17 cell levels over time occurred. There was a slight negative correlation between IL-17 values and the number of ASD which could be indicative for an appropriate seizure control within all dogs with IE. In mouse models, epileptic seizures per se have been shown to elicit an inflammatory response with an increase of cytokine levels [68–71]. Since there was no correlation between the last seizure event and the blood examination in the current study, the elevation of Th17 cells as well as IL-17 was interpreted as a seizure independent finding. It is still to discuss, how Th17 cells as well as IL-17 could have modified the disease of the central nervous system (CNS), how they might have crossed from blood to CNS or whether they might have originated from CNS. Lymphocytes for example, are able to cross the BBB (blood-brain barrier) via different pathways, mainly through the choroid plexus [72–75], or Th17 cells reached the brain through fenestrated regions under physiological conditions and were activated subsequently [76, 77]. Different transport mechanisms for cytokines are described [78–80] and IL-17 could be produced by Th 17 cells, but also other cells like CNS-resident NK cells [81], resident microglia [82, 83] and astrocytes [83, 84]. Chronic IL-17 production could lead to chronic activation of microglia or astrocytosis, which in turn can lead to synaptic dysfunction [85, 86]. Accordingly, IL-17 itself might lead to disease or through IL-17 activated cells or Th17 cells themselves. Pathophysiological changes might be induced by other cytokines that are produced by Th17 cells like IL-8, IL-10, IL-21, IL-22, IL-25 and IL-26 [15, 16]. Therefore, it would be of great interest to measure these other cytokines in dogs with IE. Additionally, in human studies in which IE was revealed as an immune-mediated disease, antibodies were detected [38–43, 65, 67, 87]. Such measurements of autoantibodies could be useful in the single cases with elevated Th17 cells. The reasons for autoimmune diseases are scattered broadly and failure of self-tolerance is still unknown. Regulatory T cells (Treg cells, CD4 + CD25 Foxp3 +) are thought to be of particular importance to regulate Th17 cells and preventing autoimmune diseases [19, 88–91]. Therefore, further investigations should include the measurement of Treg cells in dogs with IE.

In summary, the hypothesis was confirmed that a Th17 cell response in some dogs with IE occurs. In these rare cases, an underlying mild sterile encephalitis could be present and it seems that these dogs have an increased risk in experiencing combined CS and StEp. In those patients, immunosuppressive or anti-inflammatory drugs could be a worthy treatment trial.

## Conclusion

In conclusion, our findings suggest that in some individual dogs with IE a Th17 cell-mediated immune response can be causative for epileptic seizures. A further step in understanding the variability of IE in dogs was provided in the current study.

## Supporting information

S1 Table Descriptive data of dogs with idiopathic epilepsy. CSF: Cerebrospinal fluid; CT: Computer tomography; IE: Idiopathic epilepsy; exam.: Examination; F: female intact; FN: female neutered; kg: kilogram; M: male intact; MN: male neutered; m.: months; MRI: Magnetic resonance imaging.
(DOCX)

S2 Table Descriptive data of healthy control dogs. F: female intact; kg: kilogram; M: male intact; m.: months.
(DOCX)

S3 Table Study data of dogs with Idiopathic Epilepsy (IE). CSF: Cerebrospinal fluid; IE: Idiopathic epilepsy.
(DOCX)

S4 Table Study data of healthy control dogs. CSF: Cerebrospinal fluid.
(DOCX)

S5 Table Study data of dogs with idiopathic epilepsy and changes of the amount of Th17 cells over time. DM1: date of measurement at time point 1; DM2: date of measurement at time point 2.
(DOCX)

## Abbreviations

A: age grade (age at seizure onset in months)
A-B: period from last seizure day to blood drawing/CSF tap
ASD: antiseizure drug
APC: Allophycocyanin
BBB: blood-brain barrier
CD: cluster of differentiation
CD3+: cluster of differentiation receptor 3 positive
CD4+: cluster of differentiation receptor 4 positive
CD8alpha+: cluster of differentiation receptor 8 alpha positive
CD11b: cluster of differentiation receptor 11b positive
CD21: cluster of differentiation receptor 21 positive
C: control group
CNS: central nervous system
CO_2_: Carbon dioxide
CS: cluster seizures
CSF: cerebrospinal fluid
D: duration of the disease (period from first seizure day to blood drawing/CSF tap)
DM1: date of measurement 1
DM2: date of measurement 2
ECVN: European College of Veterinary Neurology
EDTA: ethylenediaminetetraacetic acid
ELISA: Enzyme-linked immunosorbent assay
et al.: et alia
FCS: fetal calf serum
Fig: figure
FITC: Fluorescein-isothiocyanate
HEPES: 4-(2-hy-droxyethyl)-1-piperazineethanesulfonic acid
IE: idiopathic epilepsy
IL: interleukin
IVETF: International Veterinary Epilepsy Task Force
kg: kilogram
MRI: magnetic resonance imaging
NK cell: natural killer cell
NSAIDs: non-steroidal anti-inflammatory drugs
PBMCs: peripheral blood mononuclear cells
PE-Cyanine 7: Phycoerythrin conjugated with Cyanine7- dye
PenStrep: 100 U/mL Penicillin-G
PMA: Phorbol-12-myristate-13-acetate
RPMI: RPMI Medium 1640 + L-Glutamine + Phenol red
RS: reactive seizures
rSpear: Spearman’s rank correlation coefficient
SE: structural epilepsy
SF/6M: mean of seizure days six months before blood drawing/CSF tap (seizure frequency)
SF/M: seizure days one month before blood drawing/CSF tap (seizure frequency)
SRMA: steroid-responsive meningitis-arteritis
SS: seizure severity
ST: seizure type
StEp: status epilepticus
Th cells: T helper cells
Th 1 cells: T helper 1 cells
Th2 cells: T helper 2 cells
Th17 cells: T helper 17 cells
TR: treatment response
Treg: T regulatory cells
