# Supplementary material for: Th17 cell-mediated immune response in a subpopulation of dogs with idiopathic epilepsy
Source: PLoS One. 2022 Jan 13;17(1):e0262285. doi: 10.1371/journal.pone.0262285 (PMC8757915; doi:10.1371/journal.pone.0262285)
Supplement: S1 Table — CSF: Cerebrospinal fluid; CT: Computer tomography; IE: Idiopathic epilepsy; exam.: Examination; F: female intact; FN: female neutered; kg: kilogram; M: male intact; MN: male neutered; m.: months; MRI: Magnetic resonance imaging. (DOCX) [file pone.0262285.s001.docx]

**S1 Table. Descriptive data of dogs with idiopathic epilepsy.**

| **Dog with IE** | **Breed** | **Gender** | **Weight (kg)** | **Age at seizure onset (m.)** | **Duration of disease (m.)** | **Performed MRI and CSF exam.** |
| --- | --- | --- | --- | --- | --- | --- |
| 1 | Crossbreed dog | MN | 52 | 9 | 20 | Yes |
| 2 | Border Collie | M | 26 | 44 | 24 | Yes |
| 3 | Tibetan Terrier | F | 10 | 55 | 1 day | No |
| 4 | Pug | F | 9 | 19 | 2 | Yes |
| 5 | Labrador Retriever | F | 36 | 58 | 2 | No |
| 6 | Crossbreed dog | M | 18 | 26 | 133 | Yes |
| 7 | Labrador Retriever | FN | 32 | 23 | 19 | MRI, no CSF |
| 8 | Miniature Schnauzer | M | 8 | 6 | 2 | Yes |
| 9 | Australian Shepherd | F | 32 | 46 | 8 | Yes |
| 10 | Australian Shepherd | F | 13 | 3 | 12 | Yes |
| 11 | Crossbreed dog | M | 26 | 60 | 53 | Yes |
| 12 | Rhodesian Ridgeback | M | 42 | 21 | 7 | Yes |
| 13 | Australian Shepherd | MN | 24 | 44 | 51 | Yes |
| 14 | Boxer | MN | 37 | 15 | 10 | Yes |
| 15 | Havanese | M | 4 | 20 | 24 | No |
| 16 | Labrador Retriever | M | 35 | 47 | 19 | Yes |
| 17 | Australian Shepherd | MN | 40 | 51 | 18 | Only CT Scan |
| 18 | Tervueren | M | 26 | 18 | 30 | Yes |
| 19 | Huskie | F | 24 | 29 | 1 day | Yes |
| 20 | Border Terrier | M | 10 | 44 | 12 | No |
| 21 | Australian Shepherd | M | 27 | 32 | 1 | Yes |
| 22 | Australian Shepherd | FN | 17 | 18 | 32 | No |
| 23 | Pekingese | MN | 9 | 60 | 25 | Yes |
| 24 | Goldern Retriever | M | 43 | 34 | 15 | Yes |
| 25 | Crossbreed dog | MN | 30 | 79 | 22 days | Yes |
| 26 | English Springer Spaniel | M | 29 | 53 | 24 | Yes |
| 27 | German Shepherd | MN | 38 | 117 | 2 | Yes |
| 28 | Crossbreed dog | M | 21 | 39 | 24 | Yes |
| 29 | Elo | MN | 23 | 79 | 37 | Yes |
| 30 | Labrador Retriever | FN | 30 | 83 | 20 | Yes |
| 31 | Labrador Retriever | MN | 31 | 36 | 80 | Yes |
| 32 | Bolonka Zwetna | FN | 4 | 81 | 18 | Yes |
| 33 | Border Collie | MN | 30 | 26 | 79 | Yes |
| 34 | Crossbreed dog | M | 13 | 24 | 129 | Yes |
| 35 | Labrador Retriever | MN | 33 | 23 | 84 | No |
| 36 | German Mittelspitz | FN | 5 | 33 | 2 | Yes |
| 37 | Wire-haired Dachshund | MN | 12 | 27 | 150 | No |
| 38 | Dalmatian | MN | 40 | 72 | 4 | Yes |
| 39 | Dogo Argentino | M | 51 | 28 | 6 | Yes |
| 40 | Belgian Shepherd | M | 38 | 24 | 67 | Yes |
| 41 | German Shorthair | FN | 26 | 35 | 12 days | Yes |
| 42 | Crossbreed dog | MN | 50 | 91 | 2 | Yes |
| 43 | English Bulldog | M | 32 | 44 | 16 | Yes |
| 44 | Boston Terrier | M | 8 | 16 | 31 | Yes |
| 45 | Landseer | FN | 68 | 43 | 55 | Yes |
| 46 | Beagle | M | 16 | 63 | 7 | Yes |
| 47 | Maltese | MN | 8 | 18 | 97 | Yes |
| 48 | Australian Shepherd | MN | 31 | 49 | 12 | Yes |
| 49 | Shiba Inu | F | 17 | 24 | 62 | No |
| 50 | Labrador Retriever | MN | 36 | 59 | 9 | No |
| 51 | Bolonka Zwetna | FN | 7 | 88 | 7 days | Yes |
| 52 | Labrador Retriever | FN | 25 | 42 | 4 | Yes |
| 53 | Maltese | M | 4 | 42 | 10 | Yes |
| 54 | Huskie | M | 28 | 16 | 48 | Yes |
| 55 | German Shepherd | FN | 31 | 69 | 36 | Yes |
| 56 | Samoyed | M | 29 | 34 | 78 | Yes |
| 57 | Crossbreed dog | MN | 10 | 84 | 50 | Yes |

CSF: Cerebrospinal fluid; CT: Computer tomography; IE: Idiopathic epilepsy; exam.: Examination; F: female intact; FN: female neutered; kg: kilogram; M: male intact; MN: male neutered; m.: months; MRI: Magnetic resonance imaging.
