# Supplementary material for: Th17 cell-mediated immune response in a subpopulation of dogs with idiopathic epilepsy
Source: PLoS One. 2022 Jan 13;17(1):e0262285. doi: 10.1371/journal.pone.0262285 (PMC8757915; doi:10.1371/journal.pone.0262285)
Supplement: S2 Table — F: female intact; kg: kilogram; M: male intact; m.: months. (DOCX) [file pone.0262285.s002.docx]

**S2 Table. Descriptive data of healthy control dogs.**

| **Healthy control dogs** | **Breed** | **Gender** | **Age (m.)** | **Weight (kg)** |
| --- | --- | --- | --- | --- |
| 1 | Beagle | M | 36 | 13 |
| 2 | Beagle | M | 24 | 12 |
| 3 | Beagle | M | 36 | 14 |
| 4 | Beagle | F | 36 | 12 |
| 5 | Beagle | M | 24 | 15 |
| 6 | Beagle | F | 24 | 15 |
| 7 | Beagle | F | 24 | 15 |
| 8 | Beagle | M | 24 | 18 |
| 9 | Beagle | M | 24 | 11 |
| 10 | Beagle | F | 24 | 14 |
| 11 | Beagle | M | 24 | 12 |
| 12 | Beagle | F | 24 | 14 |
| 13 | Beagle | M | 24 | 12 |
| 14 | Beagle | M | 24 | 15 |
| 15 | Beagle | M | 36 | 12 |

F: female intact; kg: kilogram; M: male intact; m.: months.
