# Supplementary material for: Th17 cell-mediated immune response in a subpopulation of dogs with idiopathic epilepsy
Source: PLoS One. 2022 Jan 13;17(1):e0262285. doi: 10.1371/journal.pone.0262285 (PMC8757915; doi:10.1371/journal.pone.0262285)
Supplement: S3 Table — CSF: Cerebrospinal fluid; IE: Idiopathic epilepsy. (DOCX) [file pone.0262285.s003.docx]

**S3 Table. Study data of dogs with idiopathic epilepsy (IE).**

| **Dogs with IE** | **Unstimulated Th17 cells/µL** | **Stimulated Th17 cells/µL** | **IL-17 pg/mL CSF** | **IL-17 pg/mL serum** |
| --- | --- | --- | --- | --- |
| 1 | 32.26 | 50.54 | - | - |
| 2 | 10.88 | 20.95 | - | - |
| 3 | 27.98 | 69.25 | - | - |
| 4 | 20.38 | 62.07 | 3.24 | 456.71 |
| 5 | 17.21 | 44.43 | - | - |
| 6 | 23.44 | 17.11 | - | - |
| 7 | 2.24 | 8.71 | - | - |
| 8 | 14.23 | 70.21 | 6.20 | 29.47 |
| 9 | 10.76 | 13.31 | 5.47 | 253.69 |
| 10 | 29.56 | 59.97 | 5.30 | 402.86 |
| 11 | 3.86 | 8.51 | 3.40 | 383.20 |
| 12 | 16.96 | 33.7 | 3.66 | 487.63 |
| 13 | 4.67 | 26.83 | 3.07 | 352.86 |
| 14 | 10.77 | 31.52 | 2.75 | 170.56 |
| 15 | 9.5 | 17.69 | 18.06 | 215.80 |
| 16 | 22.9 | 30.39 | 5.45 | 484.87 |
| 17 | 27.49 | 29.16 | - | - |
| 18 | 11.35 | 30.81 | - | - |
| 19 | 19.42 | 22.08 | 4.54 | 160.64 |
| 20 | 9.22 | 29.96 | - | - |
| 21 | 5.98 | 32.39 | 4.35 | 151.42 |
| 22 | 5.99 | 12.73 | - | - |
| 23 | 9.78 | 23.48 | - | - |
| 24 | 17.24 | 46.57 | 3.57 | 136.39 |
| 25 | 6.1 | 28.62 | 3.08 | 138.55 |
| 26 | 1.84 | 2.54 | 3.11 | 334.18 |
| 27 | 6.53 | 14.4 | 4.60 | 196.56 |
| 28 | 6.84 | 70.96 | - | - |
| 29 | 2.18 | 38.61 | 2.42 | 388.90 |
| 30 | 16.61 | 61.12 | 2.99 | - |
| 31 | 13.94 | 43.16 | 3.38 | 110.68 |
| 32 | 20.35 | 59.86 | 5.26 | 166.64 |
| 33 | 17.36 | 53.99 | 4.83 | - |
| 34 | 23.51 | 59.09 | - | - |
| 35 | 10.32 | 48.45 | - | - |
| 36 | 14.49 | 39.25 | - | - |
| 37 | 8.42 | 81.71 | - | - |
| 38 | 4.56 | 39.76 | 6.22 | 93.88 |
| 39 | 12.08 | 50.84 | 3.14 | 245.66 |
| 40 | 8.43 | 58.08 | 2.37 | 124.44 |
| 41 | 6.43 | 47.83 | 4.00 | - |
| 42 | 9.62 | 92.8 | 3.86 | 130.05 |
| 43 | 21.78 | 76.1 | 3.25 | 88.08 |
| 44 | 4.92 | 41.57 | 3.55 | 333.10 |
| 45 | 11.11 | 76.37 | 4.67 | 109.97 |
| 46 | 5.24 | 24.76 | 4.79 | 82.79 |
| 47 | 6.34 | 51.21 | 6.73 | 408.98 |
| 48 | 21.53 | 120.75 | 2.58 | 64.62 |
| 49 | 21.86 | 114.67 | - | - |
| 50 | 40.57 | 144.42 | - | - |
| 51 | 75.41 | 175.75 | 6.76 | 1001.00 |
| 52 | 2.35 | 135.79 | 5.82 | 158.63 |
| 53 | 25.67 | 172.73 | - | - |
| 54 | 13.43 | 114.25 | - | - |
| 55 | 37.16 | 138.00 | - | - |
| 56 | 48.04 | 280.74 | - | 908.71 |
| 57 | 32.07 | 180.57 | 4.31 | 1001.00 |

CSF: Cerebrospinal fluid; IE: Idiopathic epilepsy.
