# Supplementary material for: Th17 cell-mediated immune response in a subpopulation of dogs with idiopathic epilepsy
Source: PLoS One. 2022 Jan 13;17(1):e0262285. doi: 10.1371/journal.pone.0262285 (PMC8757915; doi:10.1371/journal.pone.0262285)
Supplement: S4 Table — CSF: Cerebrospinal fluid. (DOCX) [file pone.0262285.s004.docx]

**S4 Table. Study data of healthy control dogs.**

| **Healthy control dogs** | **Unstimulated Th17 cells/µL** | **Stimulated Th17 cells/µL** | **IL-17 pg/mL CSF** | **IL-17 pg/mL serum** |
| --- | --- | --- | --- | --- |
| 1 | 40.96 | 87.03 | 2.53 | 155.06 |
| 2 | - | - | 2.86 | 34.77 |
| 3 | 1.96 | 25.18 | 8.76 | 78.75 |
| 4 | - | - | 3.04 | 63.16 |
| 5 | 30.28 | 54.91 | 2.00 | 72.93 |
| 6 | - | - | 0.10 | 75.58 |
| 7 | 17.16 | 47.78 | 0.10 | 14.99 |
| 8 | 10.33 | 24.90 | 2.64 | 35.52 |
| 9 | - | - | 0.10 | 105.58 |
| 10 | - | - | 2.53 | 722.27 |
| 11 | 18.35 | 47.94 | - | - |
| 12 | 16.20 | 31.62 | - | - |
| 13 | 14.29 | 49.12 | - | - |
| 14 | 48.20 | 75.94 | - | - |
| 15 | 12.23 | 25.68 | - | - |

CSF: Cerebrospinal fluid
