# Supplementary material for: Th17 cell-mediated immune response in a subpopulation of dogs with idiopathic epilepsy
Source: PLoS One. 2022 Jan 13;17(1):e0262285. doi: 10.1371/journal.pone.0262285 (PMC8757915; doi:10.1371/journal.pone.0262285)
Supplement: S5 Table — DM1: date of measurement at time point 1; DM2: date of measurement at time point 2. (DOCX) [file pone.0262285.s005.docx]

**S5 Table. Study data of dogs with idiopathic epilepsy and changes of the amount of Th17 cells over time.**

| **DM 1: Unstimulated Th17 cells/µL** | **DM 2: Unstimulated Th17 cells/µL** | **DM 1: Stimulated Th17 cells/µL** | **DM 2: Stimulated Th17 cells/µL** |
| --- | --- | --- | --- |
| 32.26 | 7.72 | 50.54 | 18.52 |
| 10.88 | 74.87 | 20.95 | 124.07 |
| 20.38 | 26.25 | 62.07 | 101.37 |
| 6.53 | 3.5 | 14.4 | 26.27 |
| 6.84 | 2.59 | 70.96 | 35.32 |
| 2.35 | 6.24 | 135.79 | 10.03 |
| 2.18 | 0.33 | 38.61 | 7.43 |
| 2 | 11.87 | 114.25 | 63.96 |
| 37.16 | 3.64 | 138 | 42.73 |
| 14.49 | 18.15 | 39.25 | 38.74 |
| 12.08 | 8.91 | 50.84 | 81.5 |
| 6.43 | 5.99 | 47.83 | 8.94 |
| 21.78 | 4.06 | 76.1 | 31.2 |
| 32.07 | 12 | 180.57 | 286.71 |
| 4.92 | 7.47 | 41.57 | 65.15 |
| 5.24 | 10.09 | 24.76 | 47.22 |
| 6.34 | 5.99 | 51.21 | 74.49 |

DM1: date of measurement at time point 1; DM2: date of measurement at time point 2
